# Turning the ′Tides on Neuropsychiatric Diseases: The Role of Peptides in the Prefrontal Cortex

**DOI:** 10.3389/fnbeh.2020.588400

**Published:** 2020-10-20

**Authors:** Dakota F. Brockway, Nicole A. Crowley

**Affiliations:** ^1^Neuroscience Curriculum, Pennsylvania State University, University Park, PA, United States; ^2^The Department of Biology, Pennsylvania State University, University Park, PA, United States

**Keywords:** prefrontal cortex, peptides, animal models, circuitry, neurons, animal behavior

## Abstract

Recent advancements in technology have enabled researchers to probe the brain with the greater region, cell, and receptor specificity. These developments have allowed for a more thorough understanding of how regulation of the neurophysiology within a region is essential for maintaining healthy brain function. Stress has been shown to alter the prefrontal cortex (PFC) functioning, and evidence links functional impairments in PFC brain activity with neuropsychiatric disorders. Moreover, a growing body of literature highlights the importance of neuropeptides in the PFC to modulate neural signaling and to influence behavior. The converging evidence outlined in this review indicates that neuropeptides in the PFC are specifically impacted by stress, and are found to be dysregulated in numerous stress-related neuropsychiatric disorders including substance use disorder, major depressive disorder (MDD), posttraumatic stress disorder, and schizophrenia. This review explores how neuropeptides in the PFC function to regulate the neural activity, and how genetic and environmental factors, such as stress, lead to dysregulation in neuropeptide systems, which may ultimately contribute to the pathology of neuropsychiatric diseases.

## Introduction

### The Prefrontal Cortex in Humans and Rodents: Executive Control Over Neuropsychiatric Disorders

The prefrontal cortex (PFC), located in the anterior portion of the frontal lobe, is responsible for several higher-order behaviors including executive function and response to emotional stimuli (Salzman and Fusi, 2010; Grossmann, 2013). The PFC of humans has been implicated in many stress-related neuropsychiatric disorders, including anxiety (Park and Moghaddam, 2017), major depressive disorder (MDD; Murray et al., 2011), post-traumatic stress disorder (PTSD; Koenigs and Grafman, 2009), and substance use disorders (Goldstein and Volkow, 2011). Importantly, the PFC is one of the brain regions most sensitive to the detrimental effects of stress (Arnsten, 2009; Kolb et al., 2012). Stress has been shown to lead to PFC dysfunction observed in various neuropsychiatric disorders. Moreover, the PFC is known to undergo profound alterations throughout development (Teffer and Semendeferi, 2012), and is one of the last areas of the cortex to develop (Fuster, 2001).

In addition to the extensive human literature, the role of the PFC in behaviors associated with stress and neuropsychiatric disease has been heavily studied using rodent and non-human primate models. Though the role of the PFC in animal models has been heavily debated, recent attempts to standardize the definition and anatomical framework of the PFC have led to increased consistency of research (for review and synthesis see Carlén, 2017; Laubach et al., 2018). The PFC is divided dorsoventrally into various subregions; the human literature often divides the PFC into the lateral PFC (Broadmann areas 9–12 and 25) and the medial PFC (Broadmann Areas 9–12 and 44–46; Grossmann, 2013), whereas the rodent literature often sub-divides the PFC into infralimbic, prelimbic, and anterior cingulate cortex (Laubach et al., 2018). Animal model-based investigations of the PFC are allowing for a greater understanding of prefrontal cortical networks.

The PFC has both complex local circuitry and connections with other brain regions (Kolb et al., 2012). The PFC is heavily connected with other regions such as the brainstem, the thalamus, the basal ganglia, and limbic system (for review and synthesis see Van Eden and Buijs, 2000; Fuster, 2001). Its well-organized reciprocal connections with the mediodorsal nucleus of the thalamus (MD) is used as a criterion for identifying the PFC in a variety of species (Ferguson and Gao, 2015). Connections between the MD and PFC have been linked with cognitive impairment observed in many different neuropsychiatric disorders (for review and synthesis see Ouhaz et al., 2018). Moreover, excitatory afferents to the PFC arise from several other brain regions including limbic areas related to emotion such as the amygdala (Porrino et al., 1981; Lowery-Gionta et al., 2018), hippocampus (Thierry et al., 2000; Dégenètais et al., 2003; Bogart and O’Donnell, 2018), and hypothalamus (Kievit and Kuypers, 1975; Jacobson et al., 1978). Afferent projections from limbic regions carry to the PFC information about internal states and motivational significance and likely play a major role in executive control over emotional behavior (LeDoux, 1993; Fuster, 2001). The PFC also sends glutamatergic projections to multiple brain regions responsible for regulating emotional behaviors (some of them reciprocal) including the amygdala (McGarry and Carter, 2017; Bloodgood et al., 2018), the bed nucleus of the stria terminalis (BNST; Crowley et al., 2016), the striatum (Stuber et al., 2011; Britt et al., 2012; Bloodgood et al., 2018), and the periaqueductal gray (Siciliano et al., 2019).

### Peptide Populations Within the Prefrontal Cortex

Neurons within the PFC express a variety of markers and neurotransmitters (Van De Werd et al., 2010). Neuropeptides are strings of amino acids connected by peptide bonds found in the central nervous system (CNS) which play a key role in modulating neural activity (see van den Pol, 2012 for review and synthesis). Unlike classical neurotransmitters (e.g., amino acid neurotransmitters such as GABA and glutamate), neuropeptides are large molecules that are stored in large dense-core vesicles. They are often co-released along with other amino acid neurotransmitters and neuropeptide release is not restricted to the synapse. Neuropeptides diffuse long distances to act on G-protein coupled receptors. Compared to fast-acting amino acid neurotransmitters the response of receptive cells to neuropeptides is slow (often several seconds to minutes), which makes the investigation of neuropeptides complex.

Peptide-expressing gamma-aminobutyric acid (GABA)-ergic neurons (which often co-release neuropeptides) in the PFC have received dense focus throughout the decades due to their strong regulation of both glutamatergic inputs to and outputs from the PFC, as well as their ability to modulate each other. This, combined with the extensive role of the PFC in stress and neuropsychiatric disorders, has led to a keen interest in their function (Northoff and Sibille, 2014; Fogaça and Duman, 2019; McKlveen et al., 2019; Ghosal et al., 2020). These neuronal populations release both GABA and their respective neuropeptides, allowing for complex regulatory control over PFC circuits though the precise dynamics of peptide transmission vs. GABA transmission are still being elucidated throughout the brain. Also, some GABA/peptidergic neurons are thought to express multiple peptides, and it is unclear under what conditions these individual (and sometimes functionally opposing) peptides are released. Despite these challenges in studying neuropeptides, recent technological advances have made it easier to investigate peptidergic transmission in various brain regions (Al-Hasani et al., 2015, 2018; Crowley et al., 2016), including detecting neuropeptide release within the PFC (Dao et al., 2019).

**Figure 1:**
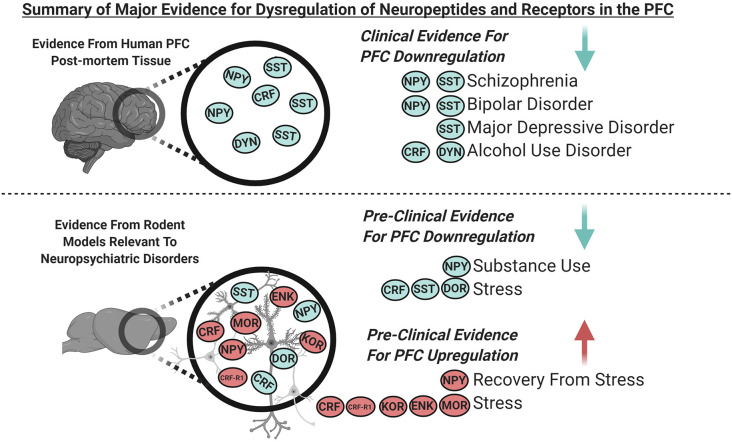
Graphical summary of the major clinical and pre-clinical findings covered in this review demonstrating dysregulated expression of neuropeptides and their receptors in the prefrontal cortex (PFC; blue indicates downregulation and red indicates upregulation). Clinical evidence from PFC of human post-mortem tissue indicates downregulation of somatostatin (SST) and neuropeptide Y (NPY) in schizophrenia and bipolar disorder, SST in major depressive disorder (MDD), and corticotropin-releasing factor (CRF) and dynorphin (DYN) in alcohol use disorder. Pre-clinical evidence from the PFC of rodent models relevant to neuropsychiatric disorders indicates that; NPY is downregulated with substance use, SST, and delta-opioid receptors (DOR) are downregulated following stress. NPY is upregulated during recovery from stress, and CRF receptor 1 (CRF-R1), kappa opioid receptors (KOR), enkephalin (ENK) and, mu-opioid receptors (MOR) are upregulated following stress. CRF is downregulated following acute stress and is increased following chronic stress.

Recent research outlined in this review sheds light on the role of diverse neuropeptides in the PFC in regulating cortical networks and controlling emotional behaviors. The current review focuses on some of the major neuropeptide populations within the PFC—notably neuropeptide Y (NPY), corticotrophin-releasing factor (CRF), somatostatin (SST), dynorphin opioids (DYN), and the endorphin/enkephalin opioid systems. Where possible, each section will explore the peptide expression and known effects, the effects of known receptors, and the role the peptide and receptors play in a variety of neuropsychiatric diseases. Importantly, this review attempts to bridge clinical studies of psychiatric populations with preclinical research investigating the neural circuit actions of PFC neuropeptides and how dysregulation of these systems contributes to specific behaviors associated with diseased states (Figure 1).

It is important to note that the profound overlap and complexity in human neuropsychiatric diseases is not well recapitulated in animal models; many animal models represent a single representation of behavior involved in disease but do not fully encompass the actual human manifestation of the disease. Disorders like MDD and substance use disorder each have many criteria for diagnosis, of which an individual only needs to meet a few characteristics. This results in great heterogeneity in the expression of neuropsychiatric disorders, and likely, great heterogeneity in underlying causes. Also, the interpretation of some animal models has evolved (for example Commons et al., 2017), further muddling the literature. While these stipulations for the interpretation of the animal literature exist, preclinical behavioral models allow for greater investigation of the peptides in context: animal models allow for a greater teasing apart of the underlying neurocircuitry, synaptic dynamics, and relationship to other peptides and neurotransmitters. These positive and negative aspects of human and animal work may be in part responsible for the lack of cohesion between the preclinical and clinical literature, discussed in greater detail throughout the review.

## Neuropeptide Y (NPY)

### Neuropeptide Y Signaling and Overall Peptide Actions

Neuropeptide Y (NPY) is a 36-amino-acid neuropeptide with potent physiological effects and dense expression in the central and peripheral nervous systems (PNS; Tatemoto et al., 1982; Adrian et al., 1983; Allen et al., 1983). It is structurally similar to peptide YY and pancreatic polypeptide and is named for its abundance of tyrosine (Y) residues (Tatemoto et al., 1982). NPY in the PNS is co-localized with noradrenaline in sympathetic nerves (Lundberg et al., 1982), and has been shown to regulate sympathetic nervous system functions such as increasing blood pressure and causing vasoconstriction (Lundberg et al., 1982; Fuxe et al., 1983).

NPY in the CNS has been shown to modulate neural activity and regulates a variety of physiological functions including feeding, mood, and memory (Fuxe et al., 1983; Wahlestedt et al., 1989; Berglund et al., 2003; Sabban et al., 2016). A growing body of evidence indicates that NPY in the CNS plays a role in the stress response (Wahlestedt et al., 1989; Heilig, 2004; Thorsell, 2008), and stress-related neuropsychiatric disorders such as anxiety, depression (Wahlestedt et al., 1989; Zukowska-Grojec, 1995; Heilig, 2004; Hou et al., 2006), and substance use disorder (Ehlers et al., 1998; Pleil et al., 2015; Robinson and Thiele, 2017). NPY has gained attention as an anti-stress peptide, it promotes resilience to stress and reduces behaviors relevant to anxiety *in vivo* (Eaton et al., 2007; Cohen et al., 2012).

NPY is considered one of the most abundant neuropeptides in the CNS (Adrian et al., 1983; Allen et al., 1983). NPY containing neuronal cell bodies and axons are found in the PFC of several different species including humans (Chan-Palay et al., 1985; Eaton et al., 2007; Robinson et al., 2019a). NPY in the cortex is known to be expressed in non-pyramidal neurons, and like other neuropeptides, is co-localized with the inhibitory neurotransmitter GABA (Hendry et al., 1984). It is often co-expressed with other peptides, namely somatostatin. Furthermore, NPY is released following physiological stimulation of NPY expressing (NPY+) GABAergic neurons in other brain regions (Li et al., 2017).

NPY+ neurons in the PFC are thought to synapse locally as well as to potentially project to other regions (Chan-Palay et al., 1985). Interestingly NPY+ neurons form connections between subregions of the PFC (Saffari et al., 2016). NPY+ neurons in the infralimbic cortex have been found to synapse onto pyramidal cells in the prelimbic cortex. NPY+ neurons in the PFC are activated by the claustrum and mediate local inhibition over pyramidal cells (Jackson et al., 2018). Due to the nature of NPY signaling both locally within the PFC and connections with other regions, NPY in the PFC is positioned as a powerful regulator over cortical networks.

The effects of the NPY peptide in the CNS are mediated primarily by five different G-protein coupled receptors, Y1, Y2, Y4, Y5, and Y6 (Pedragosa-Badia et al., 2013), though like for many peptides, off-target effects have not been completely ruled out. The Y6 receptor is non-functional in humans and rats but is functional in mice (Starbäck et al., 2000). The function of NPY in the CNS has been most well-characterized on G_i/o_-coupled Y1 and Y2 receptors (Kopp et al., 2002; Kash and Winder, 2006; Gilpin et al., 2011; Robinson and Thiele, 2017). The action of NPY is both cell and receptor-specific, for example, activation of Y1 but not Y5 receptors results in a rise in intracellular calcium in smooth muscle cells (Pons et al., 2008). In other regions, Y1 receptors act predominately postsynaptically, and Y2 receptors act predominately presynaptically.

NPY receptors in the PFC are found on both pyramidal neurons and GABAergic neurons and correspondingly, NPY has been shown to alter both inhibitory and excitatory signaling onto pyramidal neurons within the PFC (Vollmer et al., 2016), indicating circuit mediated effects. Vollmer et al. (2016) found that bath application of 1 μM NPY increases GABA_A_ receptor-mediated inhibitory postsynaptic currents (IPSCs) and a decrease in evoked α-amino-3-hydroxy-5-methyl-4-isoxazolepropionic acid receptor (AMPAR)-mediated excitatory postsynaptic currents (EPSCs) onto layer 5 pyramidal cells in the infralimbic cortex. Collectively, this suggests that NPY may function to reduce action potential discharge of pyramidal neurons of the PFC. Moreover, NPY has been shown to increase the release of other neurotransmitters in the PFC such as dopamine (Ault and Werling, 1998). The physiological effect of NPY in the PFC likely depends on several factors, such as the postsynaptic cell, receptor subtype, and locus of action. More work is needed to provide a cohesive understanding of NPY’s neuromodulatory role in the PFC.

### Stress and Post Traumatic Stress Disorder (Pre-clinical Evidence)

Pre-clinical rodent studies indicate that the effect of stress on NPY expression in the PFC is dependent on multiple factors, including the duration and type of stressor, the length of recovery, sex, and genotype. One study in Male Long Evans rats exposed to an acutely stressful event consisting of electric foot shocks found no significant change in NPY peptide after 7 days of recovery (Schmeltzer et al., 2015). Further, Schmeltzer et al. (2015) found that rats subjected to 7 days of chronic variable stress procedures, which also included foot shocks, showed no significant differences in NPY concentration in the PFC after 7 days of recovery. A separate study, also using male Long Evans rats subjected to 7 days of chronic variable stress, demonstrated no change in PFC NPY peptide 16 h following chronic variable stress; however, unlike the previous study, they found significantly increased NPY peptide in the PFC after 7 days of recovery (McGuire et al., 2011), suggesting NPY may be involved in adaptive responses to stress. However, a study using a longer duration of stress found that male Sprague–Dawley rats exposed to 36 days of a chronic unpredictable stress paradigm and 1 day of recovery demonstrated significantly reduced NPY mRNA in the PFC (Banasr et al., 2017). Together these studies suggest that the effect of stress on NPY may be dependent on the duration of the stressor, and specific dynamics of the stress procedure, including post-stress recovery time before data collection. It is possible that NPY is initially downregulated following long durations of stress but becomes upregulated during recovery as an adaptation, and more comprehensive studies are needed to confirm this hypothesis. Importantly, it has yet to be fully explored how dysregulation in NPY expression following stress exposure may be a positive or negative adaptation to further stress.

Sex hormones may also play a role in how stress affects NPY expression. A recent study explored NPY expression in the PFC in male, female, and ovariectomized female C57BL/6 mice exposed to 21 days of a chronic variable stress paradigm and 3 days of recovery (Karisetty et al., 2017). Karisetty et al. (2017) found increased NPY mRNA in the PFC in females but not in males or ovariectomized females—highlighting the increasing need for investigations into the role of sex differences. Taken together, these studies indicate NPY in the PFC is largely reduced during stress, whereas it becomes upregulated during recovery. However, the effect of stress on NPY in the PFC seems to be sex-dependent. Stress may be a contributing factor to the NPY pathology observed in neuropsychiatric disorders, and more studies are needed to understand how stress and recovery interact to regulate NPY expression in both males and females.

PTSD is a neuropsychiatric disorder that often occurs in individuals who have witnessed or experienced a traumatic or stressful event [American Psychological Association (APA) (2013)]. Symptoms of PTSD include intrusive thoughts, avoidance of reminders of a traumatic event, alterations in cognition and mood, alterations in arousal, and reactivity. Given the interaction between stress and NPY, NPY may play a role in behaviors associated with PTSD. McGuire et al. (2011) found that the increase in NPY after 7 days of chronic variable stress and 7 days of recovery in rats was associated with exaggerated fear response and recall. Also, NPY infused into the infralimbic region of the PFC in male Sprague–Dawley rats inhibits the consolidation of extinction, resulting in impaired retrieval of extinction memory *via* the Y1 receptor (Vollmer et al., 2016). In short, these preclinical findings indicate that increased NPY may contribute to fear-related behaviors associated with PTSD.

Human subjects with PTSD demonstrate decreased NPY in the cerebrospinal fluid (CSF; Sah et al., 2009, 2014). It is unknown how NPY regulation is changed in the PFC of PTSD subjects. Pre-clinical findings from Vollmer et al. (2016) would suggest increased NPY expression in the PFC of PTSD patients however clinical studies are needed to confirm this hypothesis. Importantly, the causes of PTSD in humans is incredibly varied—ranging from life events (Simon et al., 2020), natural disasters (Cénat et al., 2020), domestic violence (Kofman and Garfin, 2020), pandemics (Kaseda and Levine, 2020) to other causes, and it is unlikely that the animal literature models the breadth of these events.

### Substance Use Disorder (Pre-clinical Evidence)

To meet the criteria for substance use disorder (including alcohol use disorder) individuals must display at least 2 of 11 symptoms ranging from impaired control over substance use, social impairment, risky behavior, and the development of tolerance and withdrawal [American Psychological Association (APA) (2013)]. Several animal studies that investigated the role of NPY in the CNS on alcohol consumption indicate that lack of NPY can promote alcohol consumption. For instance, NPY knockout mice exhibit increased alcohol consumption (Thiele et al., 1998, 2000; Robinson and Thiele, 2017). Recently, the relationship between NPY in the medial PFC (mPFC) and binge alcohol consumption was explored in male and female C57BL/6J mice using the drinking in the dark paradigm (Robinson et al., 2019a). Robinson et al. (2019a) found that binge drinking reduced NPY immunoreactivity in the mPFC. Also, Robinson et al. (2019a) discovered opposing effects of Y1 and Y2 receptors in the mPFC, consistent with the literature on NPY and drinking elsewhere in the brain, such as the BNST (Kash et al., 2015). Robinson et al. (2019a) found that separate activation of Y1 receptors and inhibition of Y2 receptors both resulted in decreased binge ethanol intake in the mPFC, suggesting that NPY may reduce alcohol consumption through activation of Y1 receptors. Because Y2 receptors are predominantly auto-receptors on NPY neurons, antagonism of the Y2 receptor may promote activation of NPY neurons and subsequent NPY Y1 receptor activation, thus the synaptic location may account for this differential effect. NPY Y1 and Y2 receptors both signal through Gi/o signaling cascades, little evidence thus far suggests differences in NPY affinity for these receptors, or in the intracellular signaling cascades at either receptor (Kash et al., 2015), thereby supporting Robinson et al.’s (2019a) conclusion that synaptic location and local circuitry are at play. Moreover, other drugs of abuse such as cocaine lead to reductions in NPY in the PFC (Wahlestedt et al., 1991). These findings indicate that NPY and its receptors play an important role in alcohol consumption, and the effect of NPY likely depends largely on the specific NPY receptor which is activated. NPY and its corresponding receptors in the PFC are hypothesized to regulate behaviors associated with substance use disorder in humans, but clinical investigations are needed to confirm this hypothesis. Importantly, NPY is downregulated by multiple forms of substance use (both binge alcohol and cocaine) in animal models.

### Major Depressive Disorder (Pre-clinical and Clinical Evidence)

MDD is characterized by a combination of symptoms including, depressed mood and loss of interest or pleasure, present during the same 2-week period [American Psychological Association (APA) (2013)]. Clinical studies indicate that NPY concentration in the frontal cortex is largely unchanged in patients with a clinical diagnosis of MDD, however, it may be involved with emotional regulation. One study found NPY was significantly lower in victims of suicide when compared to accidental-death control subjects (Widdowson et al., 1992) suggesting that NPY deficits in this region may be linked with emotional regulation and depression. However, a subsequent study did not support a role for NPY in MDD (Ordway et al., 1995). When comparing victims of suicide with a co-occurring diagnosis of MDD to accidental-death controls with no diagnosed psychiatric disorders, there was no significant difference in NPY concentration (Ordway et al., 1995). Consistent with this result, another study in humans found no change in the levels of PFC NPY in subjects diagnosed with MDD (Kuromitsu et al., 2001). The relationship between MDD and suicide is complex and remains to be fully elucidated, particularly in terms of causality, therefore while NPY may play a role in emotional regulation, it appears largely unaffected in the clinical population diagnosed with MDD. Further, there were no significant differences in prefrontal Y1 or Y2 receptor mRNA between control and MDD subjects (Caberlotto and Hurd, 2001). Together, clinical studies indicate that in humans diagnosed with MDD, expression NPY and NPY Y1 and Y2 receptors in the PFC are largely unchanged.

Genes regulating NPY have been shown to interact with environmental factors such as stress to increase susceptibility to negative emotional symptoms associated with anxiety and depression (Sommer et al., 2010). Sommer et al. (2010) identified a variant allele in the NPY promoter which results in increased NPY mRNA in the anterior cingulate cortex subregion of the PFC. This variation in the NPY gene increases susceptibility to stress and may contribute to the symptoms of depression. This finding in humans links genetic regulation of NPY with emotional symptoms following stress, however, it does not provide direct evidence for the role of NPY in stress or MDD.

Despite these clinical findings, pre-clinical rodent models exhibiting behaviors associated with depression point towards decreased NPY in the PFC. Both types of NPY mRNA were found to be downregulated in the PFC of a rat model of depression (Flinders Sensitive Line, FSL) when compared to controls (Flinders Resistant Line, FRL; Melas et al., 2012). The FSL is a selectively bred rat line that partially resembles the behavior of depressed individuals and exhibits other neurochemical changes associated with depression (Overstreet et al., 2005). Another model using an intraperitoneal injection of lipopolysaccharide (LPS) to induce depressive-like behavior in Sprague–Dawley rats found decreased NPY and Y2 receptor expression in the mPFC (Wang et al., 2019). It is important to note that these models do not fully capitulate the disease pathology observed in humans with MDD, and their construct, face, and predictive validity must be assessed when comparing these models with clinical populations.

Preclinical studies using rodent models are helping to investigate the potential of NPY to modulate behaviors associated with depression. Wang et al. (2019) also found that NPY itself can reduce depressive-like behavior (as measured by open field test and sucrose preference test) in LPS treated rats when administered in the PFC. Local infusion of NPY into the mPFC reduced LPS-induced depressive-like behaviors in both the open field test and sucrose preference test in Sprague–Dawley rats. This effect was determined to be mediated by the Y2 receptor, as PFC administration of the Y2 receptor antagonist abolished, and administration of Y2 receptor agonist mimicked, antidepressant-like behavioral effects of NPY. However, a different study in male Sprague–Dawley rats found that NPY infusion into the infralimbic cortex subregion of the mPFC did not affect depression-like behavior in the forced swim test (Vollmer et al., 2016). As interpretations of behavior in the forced swim test continue to evolve (Commons et al., 2017), NPY may affect a specific subset of behaviors associated with depression such as the behaviors measured by the open field test and sucrose preference test and not affect other types of behaviors such as those measured by forced swim test, and more studies are needed to determine the specific behavioral effect of NPY. Another possibility is that the effect of NPY in the PFC is region-specific even within the PFC, and its effect may depend largely on the site of injection. The pre-clinical work demonstrates support for the hypothesis that NPY plays a role in regulating a specific subset of behaviors associated with depression, and further pre-clinical and clinical studies are needed to fully examine this hypothesis.

### Schizophrenia and Bipolar Disorder (Pre-clinical and Clinical Evidence)

Many neuropsychiatric disorders have been linked with reductions in PFC NPY, suggesting that it plays an essential role in regulating emotional behaviors. Notably, NPY in humans is found to be altered in schizophrenia, and bipolar disorder (Wu et al., 2011). Schizophrenia is characterized by symptoms such as delusions, hallucinations, social and occupational dysfunction [American Psychological Association (APA) (2013)]. Bipolar disorder is characterized by extreme emotional states that occur at distinct times called mood episodes. These mood episodes are often characterized as manic, or depressive. Multiple studies have observed expression deficits in NPY and NPY mRNA in the PFC from post-mortem tissue of subjects with schizophrenia and subjects with bipolar disorder (Gabriel et al., 1996; Kuromitsu et al., 2001; Hashimoto et al., 2008a), although it is important to note that Caberlotto and Hurd (1999) found decreased NPY mRNA only in post mortem tissue of subjects with a clinical diagnosis of bipolar disorder, and not those with schizophrenia or other disorders. Moreover, NPY Y1 and Y2 receptor mRNA in the PFC was unaltered in post mortem tissue of subjects with bipolar disorder, and schizophrenia when compared to healthy controls (Caberlotto and Hurd, 2001). Male rats treated with Lithium exhibited increased NPY-like immunoreactivity in the frontal cortex, suggesting that NPY may be involved in the response to treatments to MDD and bipolar disorder. Together these findings indicate that deficits in NPY expression in the PFC are observed in both schizophrenia and bipolar disorder.

## Corticotropin-Releasing Factor (CRF)

### CRF Neuropeptide Signaling and Overall Actions

Corticotropin-releasing factor (CRF) also known as Corticotropin-releasing hormone (CRH; referred to here as CRF) is a 41-amino acid neuropeptide which belongs to a family of neuropeptides including Urocortin 1 (Vaughan et al., 1995), Urocortin 2 (Reyes et al., 2001), and Urocortin 3 (Lewis et al., 2001). CRF was first characterized by hypothalamic extracts for its ability to stimulate the release of corticotropin and beta-endorphin from rat anterior pituitary cells *in vitro* (Vale et al., 1981). CRF is highly expressed in the paraventricular nucleus of the hypothalamus (Swanson et al., 1983) where it acts to activate the primary stress response pathway or hypothalamic pituitary adrenal (HPA) axis by promoting the release of stress hormones such as glucocorticoids and cortisol from the adrenal gland (Turnbull and Rivier, 1997; Bale and Vale, 2004; Dedic et al., 2017).

In addition to its role in the hypothalamus, CRF acts in other regions of the CNS where it functions to robustly modulate circuit function and to regulate behaviors associated with stress and addiction (Koob and Heinrichs, 1999; Kash and Winder, 2006; Orozco-Cabal et al., 2006; Silberman et al., 2013). CRF is widely distributed throughout the mammalian brain and is highly expressed in the PFC of mammals including humans (Pandey et al., 2019), rats (Swanson et al., 1983), and mice (Chen et al., 2020). CRF expressing neurons (CRF+) are found in the PFC and are predominately in layers II and III (Swanson et al., 1983). CRF+ neurons in the PFC are a subclass of inhibitory neurons, a large portion of which also express vasoactive intestinal polypeptide (VIP) or calretinin (Chen et al., 2020). Importantly, CRF+ neurons in the PFC become active during stress (Chen et al., 2020) and withdrawal (George et al., 2012). Activation of PFC CRF+ neurons results in local CRF release to modulate cognition and behavior (Hupalo et al., 2019b). Direct administration of CRF into the PFC results in impaired working memory, and CRF antagonism improves working memory, indicating that CRF acts in the PFC to regulate cognitive behaviors (Hupalo and Berridge, 2016). The dysfunction of CRF in the CNS occurs in stress-related disorders including PTSD, MDD, and anxiety (Hupalo et al., 2019a). Taken together, these findings suggest an important interaction between stress and CRF release which may contribute to neuropsychiatric disease.

The action of CRF is exerted through two major G protein-coupled receptors subtypes: CRF-R1 and CRF-R2 (Lovenberg et al., 1995; Perrin et al., 1995; Grammatopoulos et al., 2001; Dautzenberg and Hauger, 2002; Hauger et al., 2003). CRF-R1 and CRF-R2 are present in the PFC, however, CRF-R2 is expressed at low levels in the PFC of rodents (de Souza et al., 1985; Millan et al., 1986; Sánchez et al., 1999; Van Pett et al., 2000). CRF receptor expression in the cortex shows a high correlation with the distribution of CRF (de Souza et al., 1985) and CRF depresses excitatory synaptic transmission in PFC slices (Zieba et al., 2008). This supports the importance of CRF as a neuromodulator in the PFC.

In other regions such as the BNST and CEA, CRF-R1 and CRF-R2 have been found to exert opposing roles on physiology and stress-induced behaviors (Liu et al., 2004; Funk and Koob, 2007; Lowery-Gionta et al., 2012; Tran et al., 2014). Given the differences between CRF-R1 and CRF-R2 in the PFC, this effect is likely consistent. For instance, CRF-R1 acts postsynaptically, while CRF-R2 acts presynaptically in the PFC (Orozco-Cabal et al., 2008). CRF-R2 is present on presynaptic terminals in the PFC which originate from CRF+ neurons in the basal lateral amygdala (BLA; Yarur et al., 2020). Activation of presynaptic CRF-R2 limits the excitatory transmission to the PFC from the basolateral amygdala. CRF has a higher affinity for CRF-R1 (Lovenberg et al., 1995) and CRF-R2 is expressed at lower levels as compared to CRF-R1 in the PFC of rodents (Van Pett et al., 2000). The opposing function of CRF receptors is similar to the opposing function of NPY-Y1 and Y2 receptors, and the ratio of these two CRF receptors may be responsible for the net effect of the peptide in that region. Therefore, dysregulation in the expression of one or both of these receptors may contribute to the dysfunction of the CRF system.

This review focuses specifically on CRF and its action in the PFC although CRF+ neurons whose cell bodies are located in the PFC also project to other regions where they release CRF. For instance, CRF+ neurons in the PFC project to the nucleus accumbens (NAc; Itoga et al., 2019) where they act to modulate behavior through activation of CRF receptors in those regions (Kai et al., 2018). It is important to note that males and females have been shown to exhibit different behavioral responses to CRF (Wiersielis et al., 2016). Despite this finding, much of the work on the role of PFC CRF has focused exclusively on male rodents, and sex differences have been understudied. Future work is warranted to examine how dysfunction in the CRF system may contribute to behaviors related to anxiety and neuropsychiatric disorders separately in males and females.

### Stress and Post Traumatic Stress Disorder (Pre-clinical Evidence)

Multiple pre-clinical studies indicate that stress impacts the expression of CRF and CRF receptors and CRF may contribute to behaviors such as cognitive deficits associated with neuropsychiatric disease. Few preclinical studies have examined the relationship between stress and CRF-R2, and most work has focused largely on CRF-R1.

The relationship between stress, CRF, and CRF receptors in the PFC depends on the condition and duration of the stressor. Rodent models demonstrate that acute stress increases CRF and CRF-R1 mRNA in the PFC. Acute restraint stress increases both CRF mRNA and CRF-R1 mRNA in the PFC of Sprague–Dawley male rats (Meng et al., 2011). Moreover, mice (C57Bl/6N and CD1) exposed to acute social defeat stress exhibited increased CRF-R1 mRNA in the cingulate, prelimbic, and infralimbic regions of the PFC (Uribe-Mariño et al., 2016). Moreover, two stress-based rodent models of PTSD-like behaviors (though rodent models of PTSD are still evolving and emerging), one which subjected male rats to a single prolonged stressor consisting of 2-h restraint and 20 min forced swim (Wang et al., 2019), and another which subjected adolescent rats to inescapable electric foot shocks (Li et al., 2015) both resulted in increased CRF-R1 in the PFC. These experiments provide pre-clinical evidence that acute stress leads to increased CRF and CRF-R1 expression within the PFC.

Animal models of chronic stress, on the other hand, demonstrate that chronic stress leads to unchanged or decreased CRF mRNA, representing a possible adaptation to repeated stress. One study found Male Sprague–Dawley rats subjected to chronic immobilization stress (3 h a day for 21 days) showed no change in PFC CRF mRNA (Chen et al., 2008). A separate study found chronic social defeat stress (10 days) in Wistar male rats resulted in decreased CRF mRNA and increased CRF-R1 mRNA in the PFC (Boutros et al., 2016). Importantly, both acute and chronic stress lead to increased expression of CRF-R1.

CRF in the PFC has been shown to regulate behaviors associated with PTSD. Infusion of CRF into the vmPFC produces avoidance of stimuli paired with a traumatic stressor (Schreiber et al., 2017). Conversely, blockade of CRF signaling *via* CRF-R1 antagonism in the vmPFC reverses avoidance of stimuli paired with traumatic stress. Furthermore, CRF-R1 activation in the PFC following acute social defeat stress results in cognitive dysfunction (Uribe-Mariño et al., 2016). This suggests that increased CRF and CRF-R1 may contribute to behaviors associated with PTSD, and future clinical studies are needed to test this hypothesis.

Taken together these pre-clinical findings suggest that acute stress results in increased CRF whereas chronic stress results in decreased CRF. Both acute and chronic stress result in increased CRF-R1. Interestingly, CRF itself positively regulates the expression of CRF-R1 in cultured neurons (Meng et al., 2011). This suggests that the release of CRF in the PFC in response stressors may correspondingly regulate expression of CRF-R1. Increased CRF-R1 following acute stress may represent a neuroadaptation to increased CRF expression in response to stress, and this may upregulation may persist despite the downregulation of CRF with chronic stress. Deviation from homeostatic levels of CRF and CRF-R1 may contribute to PFC neurological dysfunction observed in neuropsychiatric disorders. Further pre-clinical studies are needed to compare the effects of acute and chronic stress on the expression of CRF, CRF-R1, and CRF-R2. Moreover, investigations into the expression of CRF and its receptors at multiple time points during a prolonged stressor may provide insight into the role of the CRF system in adaptation to stress.

### Anxiety and Depression (Pre-clinical and Clinical Evidence)

Clinical and pre-clinical studies both point towards a role for CRF and its receptors in anxiety and depression (for review and synthesis see Owens and Nemeroff, 1993). It has been well validated that the concentration of CRF is increased in the CSF of depressed patients and suicide victims. In the PFC, pre-clinical evidence indicates increased CRF-R1 receptor expression in response to both acute and chronic stress. Given the connection between stress and depression this effect might suggest that the clinical literature would show an increase in CRF receptor expression in humans with symptoms of depression, however, this is paradoxically not the case. Clinical evidence indicates that suicide victims exhibit a reduced density of CRF receptors in the frontal cortex as evidenced by a 23% reduction in CRF binding sites in brain tissue compared to healthy controls (Owens and Nemeroff, 1993). There are various possible reasons for these discrepancies. Importantly, the relationship between stress and suicide is not well established (i.e., not all suicide is precipitated by clear stressors, and not all stress leads to suicide). Also, Owens and Nemeroff (1993) did not distinguish between CRF-R1 and CRF-R2, and CRF-R2 is known to be less prominent in rodents than in primates. Further clinical studies are needed to determine the expression of CRF, CRF-R1, and CRF-R2 in the PFC of patients with depression. Most pressing, is that post-mortem evidence from victims of suicide does not encompass the large range of symptomology of depression, and investigation of other categories, such as MDD, is important for comparison to the animal literature.

Pre-clinical animal models also provide support for the role of PFC CRF in behaviors associated with anxiety and depression. Microinjection of CRF (0.02 μg) into the mPFC increased anxiety-like behavior in the elevated plus-maze (EPM) in both acute and chronically stressed rats (Jaferi and Bhatnagar, 2007). Interestingly, in unstressed rats, microinjection of a larger amount of CRF (0.2 μg) into the frontal cortex reduced anxiety-like behavior in the EPM. One study using various dosages of CRF in unstressed male Wistar rats found that CRF exerts opposing effects on anxiety-related behavior in the EPM depending on dose (Ohata and Shibasaki, 2011). CRF microinjected into the mPFC increases anxiety-like behavior in the EPM at lower doses (0.05 μg) and reduces anxiety-like behavior at higher doses (1.0 μg). Together, these studies reveal that CRF acts to modulate behavior associated with anxiety and depression as measured by the EPM in rodents, and the directionality of this effect may be in part, state-dependent and in part, dose-dependent.

The effect of CRF on anxiety-like behavior depends on the activation of CRF-R1. CRF microinjected into the mPFC of male Swiss mice increased anxiety-like behavior in the EPM and importantly, when a CRF-R1 antagonist was microinfused before CRF, the effect of CRF on anxiety-like behavior was blocked (Miguel et al., 2014). This study indicates that the effect of CRF on anxiety-like behavior is dependent on the CRF-R1. A separate study in male CD1 mice exposed to a live predator demonstrated that infusion of a CRF-R1 specific agonist into the mPFC reduced anxiety-like defensive behaviors including avoidance and freezing (Pentkowski et al., 2013). This supports the hypothesis that activation of CRF-R1 within the PFC regulates various behaviors associated with anxiety. CRF *via* activation of CRF-R1 in the PFC has also been shown to regulate anxiety-related behaviors through the sensitization of serotonin 5-hydroxytryptamine receptor subtype 2 (5-HT2R) signaling (Magalhaes et al., 2010), indicating a possible interaction between CRF and neurotransmitters such as serotonin. Collectively, these studies indicate that CRF plays a dual role in modulating anxiety-like behaviors through the activation of CRF-R1. Deviation from homeostatic levels of CRF may contribute to the pathology of anxiety and depression, and future clinical studies are needed to confirm this hypothesis.

### Substance Use Disorders (Pre-clinical and Clinical Evidence)

CRF systems in the brain become activated by stressors including excessive drug use, and dysfunction of CRF contributes to negative emotional states associated with withdrawal and addiction (Koob, 2013; McReynolds et al., 2014; Zorrilla et al., 2014). The human literature points towards altered CRF and its receptors in the PFC as playing a role in various substance use disorders. Post-mortem tissue from individuals with alcohol use disorder exhibit significantly decreased CRF, CRF-R1, and increased CRF-R2 mRNA in the PFC (Gatta et al., 2019). Genetic variation in CRF receptors also contributes to increased maladaptive substance use. Human genetic variation in CRF-R1 (rs110402) has been shown to interact with stress to modulate alcohol consumption and PFC activity (Glaser et al., 2014). Overall, the clinical data support a role for CRF, CRFR-1, and CRFR-2 in the PFC in substance use disorders specifically alcohol use disorder, and genetic variation in this system may contribute to the pathology.

Pre-clinical animal models also demonstrate that drug use can alter CRF expression through various mechanisms. Chronic nicotine use decreases CRF mRNA in the PFC of rodents (Carboni et al., 2018). Male Sprague–Dawley rats exhibited decreased CRF mRNA following chronic nicotine exposure (0.4 mg/kg intraperitoneal once daily for 5 days) while CRF was unchanged following acute administration of nicotine (0.4 mg/kg intraperitoneal once daily for 1 day; Carboni et al., 2018). No change in CRF mRNA was observed following 3 cycles of binge drinking in male C57BL/6J mice, however, these mice exhibited decreased CRF binding protein in the PFC (Ketchesin et al., 2016). CRF binding protein (CRF-BP) is expressed in the PFC and binds CRF with a high affinity to regulate the activity of CRF receptors (Ketchesin et al., 2017). On the other hand, heroin self-administration was not associated with alterations in CRF mRNA or CRF-BP mRNA in male Sprague–Dawley rats (McFalls et al., 2016). The variability in these results indicates that the relationship between drug use and altered CRF depends on several factors including the pharmacology of the drug in question, and the duration of drug use or abuse. Also, the difference may emerge based on whether the drug was experimenter-administered (i.e., intraperitoneal) or consumed by choice. Moreover, CRF activity can be regulated independently by other factors such as CRF-BP.

Alterations to CRF receptors in the PFC observed in humans with alcohol use disorder (Gatta et al., 2019) are also associated with various forms of substance use in rodents. Deficits in CRF are characteristic of high drinking alcohol-preferring male rats—for example, Ehlers et al. (1992) note decreased CRF concentration in the PFC of these rats. Besides these rats have been demonstrated to have increased cortical activity in the frontal cortex following CRF administration, suggesting that CRF receptors in this region may also be dysregulated (Ehlers et al., 1992). Increased heroin self-administration is associated with increased CRF-R1 in the PFC in male Sprague–Dawley rats (McFalls et al., 2016). CRF-R1 antagonism in the PFC reduced impulsivity and resulted in profound reductions in binge motivated alcohol drinking in male and female rats who had undergone early life maternal separation (Gondré-Lewis et al., 2016). Both chronic nicotine (Carboni et al., 2018) and repeated cocaine exposure (Orozco-Cabal et al., 2008) resulted in increased CRF-R2 expression in the PFC in male Sprague–Dawley rats.

CRF receptors have been shown to differentially regulate ethanol use behavior. In other brain regions such as the central extended amygdala (CEA) CRF-R1 (Lowery-Gionta et al., 2012) and CRF-R2 (Funk and Koob, 2007) play opposing roles in ethanol consumption. Substance use can modulate excitatory BLA inputs to mPFC through activation of presynaptic CRF-R2 (Orozco-Cabal et al., 2008). Orozco-Cabal and colleagues demonstrated that chronic cocaine results in increased functionality of presynaptic CRF-R2 and loss of postsynaptic function of CRF-R1 in the PFC of male rats. Moreover, an interesting, recent study found that inhibition of CRF-R2 and separate activation of CRF-R1 in the PFC both resulted in decreased binge-like ethanol consumption in male and female C57BL/6J mice, confirming that much like in the CEA, these two receptors may play opposite roles in substance use (Robinson et al., 2019b). In this work, Robinson et al. (2019b) demonstrated that co-administration of CRF-R1 and CRF-R2 antagonists attenuated the behavioral effect of CRF-R1 antagonist. This suggests that decreased binge-like ethanol drinking resulting from inhibition of CRF-R1 may result from increased activation of the CRF-R2, providing strong evidence in support of an important role of both CRF-R1 and CRF-R2 in the PFC in regulating substance abuse. However, in separate work, blocking CRF-R2 in the PFC partially inhibited cocaine-primed reinstatement of cocaine conditioned place preference (Guan et al., 2014). Overall, these findings highlight how different substances may differentially affect CRF and its receptors. These pre-clinical finding along with the clinical finding from Gatta et al. (2019) suggest that CRF-R1 and CRF-R2 may play opposing roles in substance use, and more studies are needed to confirm this hypothesis.

## Somatostatin (SST)

### SST Neuropeptide Signaling and Overall Actions

Somatostatin (SOM or SST), also known as somatotropin releasing inhibitory factor (SRIF; referred to here as SST), was characterized over 50 years ago as a hypothalamic extract capable of inhibiting the release of growth hormone from the rat anterior pituitary *in vitro* (Krulich et al., 1968). Somatostatin was originally described as a 14 (SST-14) amino acid peptide (Brazeau et al., 1973). Later, a second N-terminally extended bioactive form consisting of 28 amino acids (SST-28) was isolated and characterized (Pradayrol et al., 1980). Both isoforms are generated from the same precursor, prosomatostatin (Benoit et al., 1990). SST exhibits diverse physiological effects such as regulation of visceral functions, and inhibition of a variety of biological processes including anterior pituitary hormone secretion, insulin secretion, glucagon secretion, immune responses, DNA synthesis, and cell division (Brown and Taché, 1981; Kumar and Grant, 2010; Eigler and Ben-Shlomo, 2014; Morisset, 2017). In short, somatostatin is known to inhibit various cellular processes such as the secretion of hormones and other secretory proteins (Benali et al., 2000; Morisset, 2017). Somatostatin has been gaining attention for its role in the CNS as a neuromodulator, and in regulating behaviors linked to stress including substance abuse and affective disorders (Liguz-Lecznar et al., 2016; Robinson and Thiele, 2020).

SST in the CNS is highly evolutionarily conserved, and expression has been observed in several different species including humans, non-human primates, and rodents (Iritani and Satoh, 1991). There is a large amount of SST expression (both SST-14 and SST-28) in the PFC (Hayashi and Oshima, 1986; Lewis et al., 1986). Also, SST+ immunoreactive neurons are present at high densities in the PFC in several non-human species including macaque monkeys (Yamashita et al., 1989). SST expression is often used to classify inhibitory GABAergic neurons which mainly synapse on the dendrites of pyramidal cells within the cortex (Melchitzky and Lewis, 2008), though they have also been shown to project onto other populations including inhibitory neurons in the PFC (Cummings and Clem, 2020). SST+ neurons in the PFC have been shown to release SST under basal or tonic conditions as well as following activation (Dao et al., 2019); therefore, changes in the number or activity of SST cells in the PFC may not only result in altered GABAergic signaling but also altered SST tone. The GABAergic properties of SST cells in the cortex are known to control network activity, and the implications of SST-specific GABAergic dysfunction on neuropsychiatric disorders have been previously reviewed (Liguz-Lecznar et al., 2016; Urban-Ciecko and Barth, 2016; Robinson and Thiele, 2020).

SST exerts its biological function by activating any of five g protein-coupled SST receptors (SST-R1 to SST-R5) which are predominately Gi/Go coupled and result in inhibition of adenylyl cyclase (Patel et al., 1994; Liguz-Lecznar et al., 2016). SST-14 and SST-28 both bind and activate SST receptors with differing affinities (for example, SST-28 exhibits a greater affinity for SST-R5 than SST-14 (Liguz-Lecznar et al., 2016). SST-14 and SST-28 have been shown to exhibit differing biological effects (Hadjidakis et al., 1986). Several SST agonists and antagonists are used clinically for the treatment of diseases such as acromegaly and neuroendocrine tumors (Rai et al., 2015).

All five somatostatin receptors have been observed by immunohistochemistry in the frontal cortex of the human brain (Kumar, 2005). SST-R1 immunoreactivity is observed in the dendrites and soma of both pyramidal and non-pyramidal cells in the frontal cortex (Kumar, 2005) and is mainly presynaptic in other regions (Liguz-Lecznar et al., 2016). SST-R2 immunoreactivity was found to be confined mainly to pyramidal cells and was abundantly expressed in dendrites and processes (Kumar, 2005). SST-R3 immunoreactivity was less predominant and was observed in pyramidal cells as well as other cells such as immune cells in the frontal cortex (Kumar, 2005). SST-R3 has been shown to exist on neuronal cilia in other regions (Liguz-Lecznar et al., 2016). SST-R4 and SST-R5 expression was observed in the dendrites (Kumar, 2005).

*In vitro* studies have demonstrated that the response of cortical neurons to SST is dependent upon the concentration and corresponding receptor activation (Delfs and Dichter, 1983). Delfs and Dichter (1983) found that in cultured rat cortical neurons low concentrations of SST-14 (100 pM–1 μM) caused an excitatory response and depolarization in neurons while at higher concentrations (10 μM–1 mM) SST-14 was more likely to have no effect or to produce an inhibitory response. SST-14 and SST-28 have also been demonstrated to exhibit opposing effects on rat cortical neurons in culture (Wang et al., 1989). Wang et al. (1989) found SST-14 increased a delayed rectifier potassium current in cortical neurons, while SST-28 reduced the current. A separate study found somatostatin applied microiontophoretically to neurons in the frontal cortex elicited a dose-dependent increase in activity and caused excitation in pyramidal cells (Olpe et al., 1980). This excitatory response was likely not a result of decreased GABAergic inhibition supporting a role for somatostatin in increasing frontal cortical activity. With the recent development of receptor-specific agonists and antagonists, there is a pressing need for rigorous region and receptor-specific investigations into the neurophysiological effects of SST. Moreover, few studies have investigated both SST-14 and SST-28, and some fail to differentiate between the two. Therefore, future studies investigating the effect of both SST-14 and SST-28 are warranted.

Results concerning the action of SST on neuronal activity are sparse, and very few experiments have been conducted in the PFC. SST+ neurons in the PFC have been shown to release somatostatin in an activity dependent manner (Dao et al., 2019), and dopamine can stimulate cortical SST release (Thal et al., 1986). The release of SST in the PFC indicates that it likely functions as an important neuromodulator in this region. A thorough investigation of SST’s pharmacological action on neurotransmission in the PFC would provide the field with a framework for understanding how SST release impacts neurotransmission and behavior, and how deficits in SST observed in neuropsychiatric disorders contributes to PFC dysregulation. Importantly, studies of this nature may bridge the gap between the literature concerning the activity of SST neurons and corresponding neuropsychiatric diseases.

### Stress (Pre-clinical Evidence)

Somatostatin mRNA and peptide in the PFC are reduced following stress in rats (Banasr et al., 2017; Li et al., 2018). Male Sprague–Dawley rats exposed to 36 days of chronic unpredictable stress and 1 day of recovery exhibited significantly decreased SST mRNA measured by quantitative polymerase chain reaction (qPCR) in the PFC when compared to home cage control (Banasr et al., 2017). A study using liquid chromatography-mass spectrometry to probe the effect of multiple stressful experiences during adolescence on a broad range of neuropeptides in the PFC and hippocampus in adulthood uncovered deficits in SST-28 following adolescent stress (Li et al., 2018). Li et al. (2018) found male Wistar Han rats that underwent the peripubertal stress protocol (including exposure to fox odor and elevated platform) from postnatal day 28–42 as well as 1 h of restraint stress before sacrifice demonstrated decreased SST-28 in the PFC. In both studies the decrease was not significant in the hippocampus, suggesting that changes in SST following stress are region specific (Banasr et al., 2017; Li et al., 2018).

Stress has also been shown to affect the levels of SST receptors in the PFC (Faron-Górecka et al., 2018). Male Wistar Han rats exposed to 7 weeks of chronic mild stress exhibited increased SST-R2 binding in the PFC following stress (Faron-Górecka et al., 2018). The literature shows conflicting results concerning the effect of stress on SST+ cell number and may depend on the sex and duration of stress. Male and female SST-tdT reporter mice exposed to 14 days of chronic unpredictable stress displayed significantly decreased SST-tdT+ neurons compared to control (Girgenti et al., 2019), while in a similar study, no significant decrease in SST cell number in male Wistar rats subjected to 9 weeks of chronic mild stress was observed (Czéh et al., 2018). The longer duration of stress and the use of only males in Czéh et al. (2018) may partially account for the different results. Studies investigating the effects of both acute and chronic stress of different durations may help to uncover how the concentration of SST changes throughout a stressor.

### Major Depressive Disorder and Bipolar Disorder (Pre-clinical and Clinical Evidence)

Ample clinical evidence points towards decreased SST in human subjects with MDD and bipolar disorder. Subjects with bipolar disorder exhibit decreased SST mRNA in the PFC (Fung et al., 2014). Post mortem tissue from subjects with MDD exhibit a significant reduction in the expression of SST mRNA (measured by qPCR) and SST precursor protein (measured by western blot for prepro-SST) in the dorsolateral PFC (Sibille et al., 2011) and subgenual anterior cingulate cortex (Tripp et al., 2011). Recent studies have linked brain-derived neurotrophic factor (BDNF) expression with altered SST. Deficits in BDNF lead to decreased SST (Du et al., 2018), and BDNF itself may be required to maintain SST gene expression (Glorioso et al., 2006). This is consistent with the similar developmental expression profile of BDNF and SST mRNA which both increases during early adolescence (Du et al., 2018) and subsequently decrease with aging (Hayashi et al., 1997; McKinney et al., 2015). Decreased SST mRNA in the PFC in human subjects with MDD is correlated with reduced BDNF mRNA (Oh et al., 2019). Oh et al. (2019) found that C57BL/6J mice exposed to 7 weeks of chronic stress (an unpredictable chronic mild stress protocol) exhibited deficits in dendritic BDNF in the PFC and this decrease in dendritic BDNF may lead to a low neurotropic supply to SST neurons. Therefore, decreased BDNF may contribute to reduced SST expression and behavioral symptoms of depression (Oh et al., 2019).

Antidepressants have also been shown to modulate SST and SST receptors in the PFC in rodents. Male Sprague–Dawley rats chronically administered the antidepressant citalopram exhibited increased SST and SST-R2 density (measured by autoradiography) in the PFC and frontal cortex respectively (Pallis et al., 2009). No change in SST in the PFC was observed after treatment with the antidepressant desmethylimipramine indicating that the effect may be dependent on the pharmacology of the antidepressant. Male Sprague–Dawley rats also showed no differences in somatostatin receptors [measured by (125i)Tyrl 1-somatostatin binding] in the PFC following acute and chronic desipramine treatment (Gheorvassaki et al., 1992). These results indicate that the pharmacology of the antidepressant may determine the effect on SST and SST receptors. Understanding the effects of antidepressants on SST may help to uncover whether changes in SST contribute to the pharmaceutical efficacy of antidepressants.

### Schizophrenia (Pre-clinical and Clinical Evidence)

SST has been well studied for its role in schizophrenia and behavior related to schizophrenia in clinical and pre-clinical studies. Multiple studies have investigated the expression of SST in schizophrenic subjects (post-mortem tissue). DNA microarray for expression of GABA-related transcripts in the dorsolateral PFC of schizophrenic subjects (post mortem tissue) and matched controls revealed a robust decrease in SST mRNA in subjects with schizophrenia (Hashimoto et al., 2008a). The difference in expression of SST from healthy controls was greater than all other transcripts analyzed including NPY, GAD67, and GABA receptor subunits. This reduction in SST was further validated using Real-time qPCR and *in situ* hybridization and was replicated in multiple different studies in human subjects using qPCR (Hashimoto et al., 2008b; Fung et al., 2014; Tsubomoto et al., 2019). SST mRNA was found to be reduced (through *in situ* hybridization histochemistry and qPCR) in the orbitofrontal cortex in subjects with schizophrenia (also post mortem tissue; Joshi et al., 2015). A subsequent study in schizophrenic subjects (post mortem tissue) found that decreased SST expression in the dorsolateral PFC in schizophrenia is confined to layers 2 through 6, and both the density of SST+ neurons and the expression of SST mRNA per neuron were reduced (Morris et al., 2008). Collectively, these studies demonstrate substantial evidence in support of decreased SST in the PFC in patients with diagnosed schizophrenia.

The levels of SST mRNA were not altered in the dorsolateral PFC of monkeys chronically exposed to antipsychotic medications (Hashimoto et al., 2008b), suggesting that administration of antipsychotics as a treatment itself is not the cause of the reduced SST mRNA in schizophrenia. Moreover Rats given a single administration of haloperidol resulted in either unchanged or increased SST mRNA in the PFC further supporting the hypothesis that the decrease in SST mRNA observed in the PFC of human schizophrenic subjects reflects the disease process and is not a byproduct of antipsychotic treatment (Sakai et al., 1995).

Differential expression of SST receptors is also seen in individuals with schizophrenia. Subjects (post mortem tissue) with schizophrenia exhibited unchanged SST-R1 but significantly decreased SST-R2 mRNA in the dorsolateral PFC, and this reduction was localized to pyramidal cells in layers 5–6 (Beneyto et al., 2012). SST-R2 expression was not affected in macaque monkeys exposed to chronically high doses of antipsychotics, or in patients on or off antipsychotics at the time of death; however, macaque monkeys exposed to low doses of the antipsychotic haloperidol (a common antipsychotic for the treatment of schizophrenia) demonstrated reduced SST-R2, suggesting the results should be interpreted cautiously (Beneyto et al., 2012).

Animal studies are providing insight into the relationship between decreased PFC SST transmission and behavioral processes disrupted in schizophrenia. A recent study used viral gene knockdown to determine the behavioral effects of SST in the PFC (Perez et al., 2019). Perez et al. (2019) found that male and female Sprague–Dawley rats which underwent viral-mediated gene knockdown of SST in the PFC exhibit behavioral deficits in the negative (social interaction test) and cognitive (reversal learning test) domains consistent with those observed in schizophrenia. In two different rodent models of schizophrenia, the MK-801 model in Long Evans rats (Murueta-Goyena et al., 2020) and the BRINP1-KO model in mice (Kobayashi et al., 2018), the number of SST+ immunoreactive neurons is decreased, although this does not necessarily represent a change in the SST peptide.

The deficits in SST and SST+ neurons observed in subjects with schizophrenia and animal models of schizophrenia may be a downstream consequence of impaired BDNF signaling. Consistent with this hypothesis, strong positive correlations between BDNF protein levels and SST mRNA levels were observed in the PFC of human subjects (post mortem tissue) with schizophrenia, suggesting that BDNF may function to regulate SST expression in the PFC (Mellios et al., 2009). This parallels the findings suggesting an interaction between BDNF and SST in MDD. SST has also been shown to be regulated by BDNF through the tyrosine receptor kinase B (trkB) receptor as evidenced by reduced expression of SST in the PFC of trkB hypomorphic mice, which express significantly lower levels of trkB (Morris et al., 2008). These studies support the hypothesis that BDNF underlies changes in SST in the PFC, and may precede changes in SST, though more work is needed to understand this relationship.

## Dynorphin

### Dynorphin Signaling and Overall Peptide Actions

Dynorphin, an endogenous member of the opioid neuropeptide family (Goldstein et al., 1979), is thought to mediate negative emotional states associated with stress, depression, and drug use withdrawal (Koob and Le Moal, 2008; Bruchas et al., 2010; Knoll and Carlezon, 2010; Hang et al., 2015). Dynorphin refers to a group of neuropeptides derived from the preprodynorphin gene including Dynorphin-A 32 amino acids (Fischli et al., 1982), which binds with high affinity to kappa opioid receptors (KORs; Chavkin et al., 1982; James et al., 1982; Kakidani et al., 1982; Hauser et al., 2005). Dynorphin and KORs are present throughout the brain and activation of this system generally promotes dysphoria, anxiety-like behavior, and behaviors associated with substance use disorders (Wee and Koob, 2010; Crowley and Kash, 2015).

KORs are G protein-coupled receptors, encoded by the oprk1 gene, which are selectively activated by dynorphin (Karkhanis et al., 2017). KORs can signal *via* multiple signaling pathways including G-i/o protein-coupled inhibition of adenylyl cyclase (Konkoy and Childers, 1993; Lawrence et al., 1995; Dhawan et al., 1996; Karkhanis et al., 2017), stimulation of inwardly rectifying potassium channels (Henry et al., 1995), activation of p38 MAPK (Bruchas et al., 2006), and activation of ERK 1/2 (McLennan et al., 2008). The effects of dynorphin/KORs on neurotransmission are variable and depend on the brain region, neuron the receptor is on, and whether the receptor is expressed pre- or post- synaptically (Karkhanis et al., 2017). In other regions of the cortex, dynorphin has been shown to act presynaptically to inhibit the release of both GABA and glutamate in the same brain region (Li et al., 2012; Crowley et al., 2016). The dynorphin/KOR system is a critical mediator of both stress response and stress-induced relapse and has been linked with the CRF system (Bruchas et al., 2010). Stress-induced CRF activation leads to dynorphin release and subsequent modulation of mood by KOR activation.

Both Dynorphin and KORs are highly evolutionarily conserved and are present in the PFC in both humans and rodents (Zamir et al., 1984a,b; Dawbarn et al., 1986; McIntosh et al., 1987; Wevers et al., 1995; Hurd, 1996; Mansour et al., 1996; Svingos and Colago, 2002). KORs predominate over other types of opioid receptors such as mu-opioid receptors (MORs) in the PFC (Lahti et al., 1989). Dynorphin neurons comprise a subset of neurons in the PFC which express pre-prodynorphin and are GABAergic (Sohn et al., 2014). Approximately one-quarter of dynorphin neurons also express the neuropeptide somatostatin (Sohn et al., 2014).

Recent studies have revealed an important neuromodulatory role for the dynorphin/KOR system in the PFC. KORs in the mPFC is thought to be largely presynaptic and are localized on axons and axon terminals (Svingos and Colago, 2002). Presynaptic KORs can regulate synaptic input from other regions onto the PFC, and activation of KORs in the PFC has been shown to negatively regulate glutamatergic synaptic transmission from the BLA (Tejeda et al., 2015). Also, Tejeda et al. (2015) demonstrated that activation of KORs in the PFC decreases the frequency of miniature EPSCs onto layer 5 pyramidal cells. This work also demonstrated that activation of KORs in the PFC also directly inhibits dopamine terminals to reduce dopamine release in the PFC. Currently, Dynorphin/KOR activation is known to reduce dopamine release and dampen glutamatergic input onto pyramidal cells in the PFC, though other effects in the PFC (both on other cell populations and other neurotransmitters and peptides) have yet to be fully elucidated.

### Stress and Anxiety (Pre-clinical Evidence)

KOR mRNA and protein are affected by stress. Repeated forced swim stress in male C57BL/6 resulted in increased expression of KOR mRNA (Flaisher-Grinberg et al., 2012) and a separate study found an increase in KOR protein in male Swiss mice exposed to repeated forced swim stress (Rosa et al., 2018a). Together these results suggest that stress, specifically forced swim stress, leads to increased KOR expression. It is unknown how stress affects dynorphin expression in the PFC and future studies are needed to test this.

Dynorphin is thought to mediate dysphoria, and broadly, promote behaviors associated with anxiety. Pre-clinical rodent models indicate that the behavioral effect of dynorphin in the PFC may depend on the region of activation. Mice subjected to chronic constriction injury of the right sciatic nerve exhibit significantly increased pro-dynorphin and KOR mRNA expression in the PFC (Palmisano et al., 2019). Studies investigating the effects of dynorphin agonists and antagonists on behaviors associated with anxiety reveal a subregion specific effect of KOR activation. Microinjections of a KOR agonist (U50, 488H) or a dynorphin derivative (E-2078) into the mPFC in rats led to place aversion in the conditioned place preference paradigm suggesting that activation of KOR is associated with aversive effects in the mPFC (Bals-Kubik et al., 1993). Consistent with this, another study using the selective KOR antagonist nor-binaltorphamine (norBNI) in male Long-Evans rats found intra-mPFC injection increased center time in the open field test, suggesting decreased defense/withdraw anxiety (Tejeda et al., 2015). Together, these findings point towards a role for dynorphin and KOR activation in the mPFC in mediating aversive states and behaviors associated with anxiety.

However, these studies did not investigate the effect in specific subregions of the mPFC. Male CD-1 mice injected with the selective KOR agonist U-69, 593 in the infralimbic cortex exhibited dose-dependent decreases in avoidance behaviors in the EPM, and defensive/withdrawal anxiety in the open field (Wall and Messier, 2000). They also exhibited evidence for enhanced memory in two separate memory tests: the EPM transfer-latency test and the Y-maze test. A subsequent study by Wall and Messier (2000) investigated the effect of blocking KOR activation selectively in the infralimbic cortex using the KOR antagonist norBNI. They found that pretreatment with one injection of norBNI in the infralimbic cortex dose-dependently increased anxiety-like behavior as well as disrupted working memory in the same behavioral tasks. Importantly, the effect on anxiety-like behavior after infusion of either a KOR agonist or antagonist in the infralimbic cortex was long-lasting, and differences in EPM behavior were observed in both studies after a 24-h delay (Wall and Messier, 2000). This finding is inconsistent with the effect of KOR observed by other researchers in the mPFC, but this discrepancy may be due to the region of injection, and the effect observed by Wall and Messier may be specific to the infralimbic subregion of the PFC, which has been shown to play opposing roles to more dorsal regions of the PFC such as the prelimbic cortex in other neuropeptides. More work is needed to understand the region-specific behavioral effect of KOR activation/inhibition in different subregions of the PFC.

### Substance Use Disorder (Pre-clinical and Clinical Evidence)

Human studies point toward a dysregulation of dynorphin/KOR systems in subjects with alcohol or substance use disorders. Prodynorphin CpG dinucleotides that overlap with SNPs were differentially methylated in the dlPFC of postmortem brains from alcohol-dependent individuals (Taqi et al., 2011) suggesting a possible role for dynorphin in behaviors associated with substance dependency. One study found prodynorphin and dynorphin (both A and B) mRNA were upregulated in the dlPFC of alcoholics (Bazov et al., 2013). A second study by Bazov et al. (2018) found prodynorphin is downregulated in the PFC of alcoholics while KOR expression itself was unchanged. The authors of both studies hypothesize that the different findings are likely because the first study was underpowered, while the second study from 55 control and 53 alcoholic subjects provided a more sufficient dataset (Bazov et al., 2018). Post mortem brains from individuals with a history of marijuana use or stimulant use had increased prodynorphin mRNA in the anterior cingulate and dorsolateral prefrontal cortices respectively, but no change was found in the brains of individuals with a history of alcohol use (Peckys and Hurd, 2001). The human findings point towards dysregulation of prodynorphin with substance use, but the directionality of this effect may depend on the specific pharmacology and consumption pattern of the drug of abuse, as well as severity of abuse.

Alcohol has been shown to regulate dynorphin in rodents. Alcohol increases the density of dynorphin expressing-cells in the mPFC in rats consuming ethanol chronically compared to water controls, as assessed by digoxigenin-labeled *in situ* hybridization histochemistry (Chang et al., 2010). Male Sprague–Dawley rats treated with alcohol at a dose of 1.5 g/kg three times for 1 day exhibited increased prodynorphin in the PFC, however, no change was detected after 5 days (D’Addario et al., 2013). Prenatal alcohol exposure can lead to increased prodynorphin mRNA in the PFC in infant rats (age not further specified; Wille-Bille et al., 2018). Like other drug use, alcohol use also causes increased expression of prodynorphin. Also, directly modulating KORs has been shown to regulate drug reward. In Male Sprague–Dawley rats which received once-daily injections of cocaine (20.0 mg/kg, intraperitoneal) for 5 days, site-specific activation of mPFC KORs exacerbated the development of behavioral sensitization and increased cocaine-evoked dopamine levels (Chefer et al., 1999). More work is needed to determine how KOR activation in the PFC influences drug reward and behaviors associated with substance use disorders.

Consistent with the human literature, pre-clinical animal studies also indicate altered dynorphin/KOR following the administration of substances of abuse other than alcohol. Acute (8 mg/kg, intraperitoneal) administration of 3,4-methylenedioxy-N-methylamphetamine (ecstasy) in male Sprague–Dawley rats raised levels of prodynorphin mRNA in the PFC and decreased levels of Dynorphin-A (Di Benedetto et al., 2006). Interestingly, Di Benedetto et al. (2006) observed no change after chronic treatment. Male Wistar rats treated with both acute and chronic morphine (8.0 mg/kg intraperitoneal, once daily for one or five consecutive days) exhibited increased KOR mRNA in the PFC (Yu et al., 2012). However, at the protein level, acute morphine treatment did not affect KORs in the PFC, while chronic morphine caused downregulated KOR protein (Yu et al., 2014). Nicotine has also been shown to lead to changes in prodynorphin expression (Carboni et al., 2016). Chronic and sub-chronic administration of nicotine led to increased expression of prodynorphin mRNA in the PFC of Sprague–Dawley rats. However, this was not observed after acute administration, and no change was found in KOR mRNA with either administration paradigm. In summary, acute ecstasy, acute and chronic morphine, and sub-chronic and chronic administration of nicotine all led to increased expression of prodynorphin. More research is needed to uncover how KOR’s and dynorphin itself is altered with drug use.

Importantly, prodynorphin and KOR mRNA were found to be unchanged in the anterior cingulate and dorsolateral prefrontal cortices of post mortem brains of subjects diagnosed with schizophrenia, bipolar disorder, or major depression, and was not associated with antipsychotic treatment or suicide (Peckys and Hurd, 2001). This points towards dynorphin having a particular sensitivity to stress and/or drugs of abuse and maybe a central part of the addiction process. Based on the postulated role of the dynorphin/KOR system in mediating negative affect (such as withdrawal from drugs of abuse), more work is needed to further characterize the role of this system in the PFC concerning substance use disorders.

## Endorphin and Enkephalin

### Endorphin and Enkephalin Signaling and Overall Peptide Actions

Endorphin and enkephalin, like dynorphin, are endogenous opioid neuropeptides that are present in the CNS (Rossier, 1988). Despite their similar behavioral effects, and pharmacological action, they are derived from distinct precursors. Endorphins (canonically β-endorphins; Bruijnzeel, 2009) are derived from pro-opiomelanocortin, and enkephalins (met-enkephalin and leu-enkephalin) are derived from proenkephalin (Rossier, 1988). Endorphins and enkephalins play a role in motivational and stress circuits and have been implicated in neuroadaptations to drug abuse (Koob and Volkow, 2016). Both endorphin and enkephalin have profound pain-relieving effects and promote euphoria (Shenoy and Lui, 2018; Hicks et al., 2019).

Endorphins, enkephalins, and their precursors are highly evolutionarily conserved and are expressed in the PFC in several species including humans and rodents (Matthews et al., 1992; Hurd, 1996; Leriche et al., 2007). To date, few studies have directly examined the behavioral or physiological function of these peptides in the PFC and instead have focused on their corresponding receptors. Endorphins and enkephalins modulate neural activity through activation of G protein-coupled (GPCR) opioid receptors (Corder et al., 2018) which are also present in the PFC (Lahti et al., 1989). Endorphins preferentially bind and activate mu–opioid GPCRs (MORs), while enkephalins are non-selective agonists with an affinity for both MORs and delta-opioid GPCRs (DORs; Corder et al., 2018). DORs have been shown to regulate anxiety-like behavior, and site-specific activation of DORs in the PFC in mice reduced anxiety-like behavior (Lahti et al., 1989). Despite this early finding, few studies have investigated DORs in the PFC, and instead, most studies have focused on the MORs, due to their more well-known neuromodulatory and behavioral effects. Future studies into the role of DORs in the PFC as well as studies examining the pharmacological effects of endorphins and enkephalins in the PFC are warranted.

MORs are expressed predominately on non-pyramidal GABAergic neurons in the frontal cortex, overlapping with enkephalin expression (Taki et al., 2000; Férézou et al., 2007). This suggests that MORs function as auto-receptors on enkephalin-expressing non-pyramidal neurons. In the PFC, activation of MORs has been shown to inhibit voltage-dependent sodium currents on non-pyramidal neurons through a PKA and PKC dependent mechanism (Witkowski and Szulczyk, 2006), an effect likely mediated by auto-receptors on non-pyramidal GABAergic enkephalin neurons. Reduced voltage-dependent sodium currents would result in decreased action potentials in GABAergic cells in the PFC, and then downstream would cause decreased inhibitory currents onto pyramidal cells. Consistent with this hypothesis, MOR agonists in other regions of the cortex have been shown to decrease GABAergic transmission and decrease inhibitory currents onto pyramidal cells (Férézou et al., 2007) and hippocampal cells (Capogna et al., 1993). These findings indicate that if this effect is consistent in the PFC, MORs may cause disinhibition of pyramidal cells whereby activation of MORs on GABAergic neurons increases pyramidal cell activity by decreasing GABAergic input onto these cells.

In addition to local disinhibition of pyramidal cells, MORs in the PFC regulates excitatory input from other regions projecting to the PFC, such as presynaptic suppression of glutamate release from the thalamus (Marek and Aghajanian, 1998; Marek et al., 2001). Importantly, MORs and DORs have been shown to synergistically interact to enhance dopamine D1 receptor-induced stimulation of adenylyl cyclase activity (Olianas et al., 2012). MORs and DORs in conjunction with their endogenous ligands are positioned to precisely coordinate neural activity both within the PFC and from other brain regions. As a result, dysregulation of this system can contribute to loss of inhibitory control over executive function and behavior as is observed in multiple psychiatric disorders (Baldo, 2016).

### Stress and Anxiety (Pre-clinical Evidence)

Preclinical animal models suggest that stress exposure is linked to reduced expression of enkephalin in the PFC. Male Wistar Han rats that underwent peripubertal stress conditions (exposure to fox odor and elevated platform across post-natal days 28–42) exhibited downregulated enkephalin mRNA in the PFC (Li et al., 2018). Ninety minutes of cold and immobilizing stress in male Wistar rats resulted in significantly decreased enkephalin immunoreactivity, however, no change was observed after 30 or 180 min (Kurumaji et al., 1987). The decreased enkephalin observed only at the 90-min time point may indicate that enkephalin plays a role in adaptation to stress.

Stress has also been shown to increase MORs and decrease DORs in the PFC. Adult Swiss mice subjected to social defeat stress exhibited increased MOR protein and decreased DOR protein in the PFC in susceptible mice (Rosa et al., 2018b). Similarly, swiss mice subjected to repeated forced swim stress exhibited increased MOR expression but reduced DOR in the PFC (Rosa et al., 2018a). Neonatal handling, which is known to increase the ability to cope with stress and to decrease anxiety-like behavior is associated with increased levels of MORs in the PFC (Kiosterakis et al., 2009). This indicates that stress may lead to higher expression of MORs and decreased expression of DORs in the PFC. The increased MOR expression observed in the PFC in response to stress may be a neuroadaptation to decreased MOR ligands such as decreased enkephalin. Future work is needed to uncover the relationship between enkephalin and MORs/DORs as well as their relationship with stress.

### Substance Use Disorder (Pre-clinical and Clinical Evidence)

The rewarding effects of drugs and the development of drug-seeking behavior involve changes in opioid peptides (Koob and Volkow, 2016). The current review will not cover the breadth of focus and attention on the modern opioid epidemic, covered in-depth elsewhere (Shipton et al., 2018; Skolnick, 2018; Marshall et al., 2019). Endorphin and enkephalin in the PFC contribute to inhibitory control over appetitive behaviors, and loss of control over these behaviors is known to occur in multiple psychiatric disorders such as substance use disorders (Baldo, 2016). Infusions of MOR agonists into the ventromedial PFC of male Sprague–Dawley rats resulted in increased appetitive motivation (Selleck et al., 2015, 2018). Moreover, male Wistar rats that binge ate a highly palatable diet exhibited increased preopiomelanocortin (endorphin precursor) in the PFC (Blasio et al., 2014). These findings point towards a role for endorphin and MORs in the regulation of consummatory behavior, and thus this system may contribute to excessive drug use and behaviors observed in substance use disorders.

Alcohol and nicotine have thus far not been found to affect endorphin or enkephalin in the PFC. Post mortem brains from human alcoholics show no changes in the levels of proenkephalin, MORs, or DORs in the PFC (Bazov et al., 2013). Nicotine treatment leads to changes in endorphin and its precursor (proopiomelanocortin) in limbic regions in mice; however, only moderate decreases in proopiomelanocortin have been observed in the PFC following chronic nicotine treatment (Gudehithlu et al., 2012). Other drugs, however, such as psychostimulants, have been shown to modulate enkephalin. Male Sprague–Dawley rats administered with amphetamine and then subsequently administered with the same dose 7 days later exhibited increased proenkephalin mRNA (Morales-Mulia et al., 2007). These findings indicate that endorphin and enkephalin are likely not directly regulated by alcohol or nicotine but may be regulated by other drugs such as amphetamines.

Also, substance use has been shown to regulate MOR functioning and this may contribute to behaviors involved with substance use disorders. In humans, long-term opiate and mixed opiate/cocaine abusers exhibit decreased midazoline receptor antisera-selected (IRAS)/nischarin, a putative I1-imidazoline receptor which regulates MOR trafficking (Keller et al., 2017). AA rats bred selectively for high alcohol consumption have a significantly greater proenkephalin mRNA and greater density of MORs in the PFC (Marinelli et al., 2000) which may contribute to increased ethanol consumption, although the connection is indirect. Rats self-administering a cannabinoid receptor agonist exhibited increased MOR levels in the PFC (Fattore et al., 2007). Taken together, these results suggest endorphin/enkephalin and MORs are differentially affected by drugs of abuse, and increases in this system may be involved with specific types of substance use. Pre-clinical studies using a consistent methodology to investigate the effects of multiple different drugs of abuse on the endorphin/enkephalin system would provide a foundation for understanding how the pharmacology of the substance may contribute to the pathology of substance use disorders. Further, clinical investigations into the expression of endorphin/enkephalin in patients with various forms of substance use disorders would help to determine how these systems are differentially affected by different substances.

## Discussion

The PFC has been shown in both the clinical and pre-clinical literature to play a vital role in many neuropsychiatric disorders. Increasing evidence has shown that GABAergic and peptidergic neurons within the PFC, responsible for the modulation of glutamatergic inputs, local circuits, and pyramidal neuron outputs, play a key modulatory role in these disorders. More work is needed to understand the role of each of these individual peptide populations. Peptide effects vary depending on the model (e.g., acute stress vs. chronic stress models, length of manipulation, animal genetic strain, and sex differences). Also, further research should address interactions between peptide populations in the PFC—both those expressed in different and in the same, GABAerigc neurons. For instance, subpopulations of neurons within the PFC express both NPY and SST, while SST and dynorphin have also been found to co-express here. This overlap is surprising given the general opposing roles of NPY and dynorphin. Also, increasing technological advancements have allowed for the greater investigation of peptide release independent of co-expressed neurotransmitters, which will allow a greater understanding of when same-neuron peptides are released (i.e., under different neuronal firing frequencies or overall length of firing). Greater assessment of the combinatorial role of these peptides will better inform disease models and treatment. Overall, the literature suggests a strong and important role of peptides in the PFC in stress and neuropsychiatric disorders.

Future work, in addition to expanding the existing depth of literature, also needs to focus on filling the gap in the literature. Specifically, much of the pre-clinical work above has been conducted in male rodents, with very little focus on females. Others have eloquently expressed elsewhere (Shansky, 2018, 2019, 2020) the importance of: (a) investigating females as a stand-alone research question, as opposed to only in contrast to males as a baseline; and (b) the unlikeliness that female rodents have more behavioral and neurobiological variability, or that this variability is due to female-dominant sex hormones. Also, standard operating procedures may help to rectify some of the discrepancies seen. For instance, the multitude of stress models, with differing lengths of stress exposure and post-stress recovery periods, likely contributes to the variable effects seen.

Moreover, while pre-clinical evidence links neuropeptide systems to behaviors relevant to a disorder, these diseases themselves are incredibly complex. For example, the DSM-5 [American Psychological Association (APA) (2013)] includes five subtypes of depressive disorders (disruptive mood dysregulation disorder, MDD, persistent depressive disorder, premenstrual dysphoric disorder, substance/medication-induced depressive disorder, depressive disorder due to another medical condition, other specified depressive disorder, and unspecified depressive disorder). Animal models of depression understandably fail to capture this complexity, and importantly, often rely only on stress (whether social or environmental stress) and/or genetic manipulations (Krishnan and Nestler, 2011). This example holds for most neuropsychiatric disorders, in that animal models representing a *single* behavioral or biological manipulation are unlikely to fully recapitulate the complexity and range of the human disorder. Therefore, it is no surprise that animal models using a signal manipulation (i.e., forced swim) do not replicate the work of these nuanced psychiatric conditions in humans. Leading researchers in the preclinical field have agreed that animal models thus far are not fulling capturing human disorders but are nevertheless a crucial component of treatment identification and testing. These researchers identified key gaps in the literature needed for the synthesis of human and animal work: “*in vivo* experiments, precision, innovation, integration and complexity, and leadership setting the tone” (Bale et al., 2019). In this article, Bale et al. make a key and important statement supporting our overall characterization of peptides in the PFC, that this preclinical work does not directly model these disorders but, “*Rather, the goal is to achieve a better understanding of an essential biological function that is key to the illness and to ensure that our level of understanding is actionable for translation.”* Therefore, the discrepancies seen in the preclinical and clinical work for some neuropeptides provide a launching point for further investigation in humans.

Overall, though some discrepancies exist in the role of specific neuropeptides in specific disorders, their importance is clear and further investigation for each of them is paramount to understanding complex disorders in humans, and for targeting both preventions and interventions. By both filling in these gaps (investigating both sexes, consistency in models, further expanding research into humans), and expanding the focus into new questions and domains overall, a greater understanding of the role of peptidergic signaling in the PFC during stress and neuropsychiatric diseases may be obtained.

## Author Contributions

DB and NC wrote the manuscript. All authors contributed to the article and approved the submitted version.

## Conflict of Interest

The authors declare that the research was conducted in the absence of any commercial or financial relationships that could be construed as a potential conflict of interest.
